# Sports-related lower limb muscle injuries: pattern recognition approach and MRI review

**DOI:** 10.1186/s13244-020-00912-4

**Published:** 2020-10-07

**Authors:** Jaime Isern-Kebschull, Sandra Mechó, Ricard Pruna, Ara Kassarjian, Xavier Valle, Xavier Yanguas, Xavier Alomar, Javier Martinez, Jaume Pomés, Gil Rodas

**Affiliations:** 1 Department of Radiology, Hospital Clinic, Universitat de Barcelona, Barcelona, Spain; 2 Department of Radiology, Hospital de Barcelona, Barcelona, Spain; 3 Medical Services, Fútbol Club Barcelona, Barcelona, Spain; 4 Elite Sports Imaging, SL, Madrid, Spain; 5 Department of Radiology, Centers Mèdics Creu Blanca, Barcelona, Spain; 6 Medicine Sport Unit, Hospital Clinic and Hospital Sant Joan de Déu, Barcelona, Spain

**Keywords:** Athletic injuries, Muscle, Magnetic resonance imaging, Return to sport, Prognosis

## Abstract

Muscle injuries of the lower limbs are currently the most common sport-related injuries, the impact of which is particularly significant in elite athletes. MRI is the imaging modality of choice in assessing acute muscle injuries and radiologists play a key role in the current scenario of multidisciplinary health care teams involved in the care of elite athletes with muscle injuries. Despite the frequency and clinical relevance of muscle injuries, there is still a lack of uniformity in the description, diagnosis, and classification of lesions. The characteristics of the connective tissues (distribution and thickness) differ among muscles, being of high variability in the lower limb. This variability is of great clinical importance in determining the prognosis of muscle injuries. Recently, three classification systems, the Munich consensus statement, the British Athletics Muscle Injury classification, and the FC Barcelona-Aspetar-Duke classification, have been proposed to assess the severity of muscle injuries. A protocolized approach to the evaluation of MRI findings is essential to accurately assess the severity of acute lesions and to evaluate the progression of reparative changes. Certain MRI findings which are seen during recovery may suggest muscle overload or adaptative changes and appear to be clinically useful for sport physicians and physiotherapists.

## Key points

- MRI is widely used for the assessment of lower limb muscle injuries.
- Muscle anatomy and distribution of connective tissues are well described.
- Several classifications of muscle injuries severities are useful in the patients’ management.
- Systematic approach to evaluate MRI findings allows categorization of acute sport injuries.
- New MRI findings during the healing process are clinically relevant.

## Background

Muscle lesions are the most common category of injuries in athletes, accounting for more than 30% of injuries in soccer players [1, 2]. Despite the high frequency of muscle injuries in elite athletes and the prime concern being minimizing days lost from sporting activities, there is still a lack of uniformity in the description, diagnostic approach, and grading of muscle injuries. Different systems based on clinical examination and radiological findings, especially magnetic resonance imaging (MRI), have been proposed [3–5]. These classification systems typically focus on the site of injury (proximal, middle, distal), the involved anatomical structure (tendon, aponeurosis, fascia, muscle fibers), the imaging features on MRI scans (edema, fiber disruption, intramuscular hematoma, tendon retraction, intermuscular fluid), and the dimensions of the lesion (cross-sectional-area, craniocaudal length of edema, area of fiber disruption, discontinuity, etc.). Although they are widely used in daily practice, a systematized approach to the interpretation of MRI findings and their exact role in the prognosis of muscle injuries in elite athletes has not been established. Also, classifying the muscle lesions by MRI is a difficult task, given that the accurate interpretation of imaging findings depends on the specific muscle involved (taking into account the specific sport and mechanism of injury) and adequately high spatial resolution of the images. Accuracy of diagnosis may be compromised if MRI scans are read by health professionals with little experience in muscle injuries or if the images are of insufficient resolution.

The purpose of this educational review is to summarize the pathophysiological mechanisms of traumatic (acute) muscle injuries and anatomical peculiarities of muscles of the lower limb, to describe a specific MRI protocol that facilitates an accurate diagnosis of lesions, to present an overview of MRI-based grading systems for the classification of muscle injuries, and to characterize MRI findings of acute injuries and the healing phase of muscle injuries. The final objective is to provide radiologists a stepwise systematic approach for the effective management of lower limb muscle injuries in elite athletes.

## Mechanisms of injury and muscle anatomical features

Muscle injuries in professional players are usually indirect injuries that occur during eccentric muscle contraction. Striated muscle tissue stands out for its great contractile function related to the connective tissue [6]. The connective tissue distribution is different in each muscle, varying in location and thickness depending on the muscle’s function. However, in addition to being a force transmitter during contraction, the connective tissue acts as a structural component in the case of fascias or raphes [7, 8]. The tendon and aponeurosis are the connective tissues that contribute mainly to the transmission of force, instead of providing structural muscle framing [8, 9]. This differentiation is of great clinical relevance in determining the significance and prognosis of muscle injuries [6].

The most frequent mechanism of lower limb muscle injuries is an indirect injury (muscle strain) [10] associated with both sprinting and stretching activities [11]. The myotendinous or the myofascial/myoaponeurotic junction that represents the structurally weakest portion of the muscle [10, 12] is usually affected. Clinically, muscle strains are characterized by a sudden onset of pain usually localized in a specific muscle compartment during a period of eccentric contraction, which, depending on severity, may immediately prevent the athlete from continuing the sports activity [11]. By contrast, blunt trauma is the most common mechanism of direct muscle injuries in sports that involve collisions such as soccer, football, or rugby. Injuries due to contusion involve isolated muscle fibers or fascias. Muscle contusions tend to manifest fewer symptoms than muscle strains. Acute or recent muscle injuries are characterized by edema, vascular engorgement, and inflammatory cellular infiltration [11].

Independent of the mechanism of injury, more than 90% of muscle injuries of the lower limb in football players affect four muscle groups (hamstrings, quadriceps, adductors, and calf muscles) [1]. Clinically, it is sometimes difficult to precisely locate the lesion, and in these circumstances MRI plays a key role. Knowledge of some anatomical characteristics of the distribution of connective tissue and the orientation of fascicles/fibers in these muscle groups [13–20] is crucial for accurately interpreting the MRI findings in the diagnosis of muscle injuries of the lower limbs.

### Hamstring complex

**Fig. 1:**
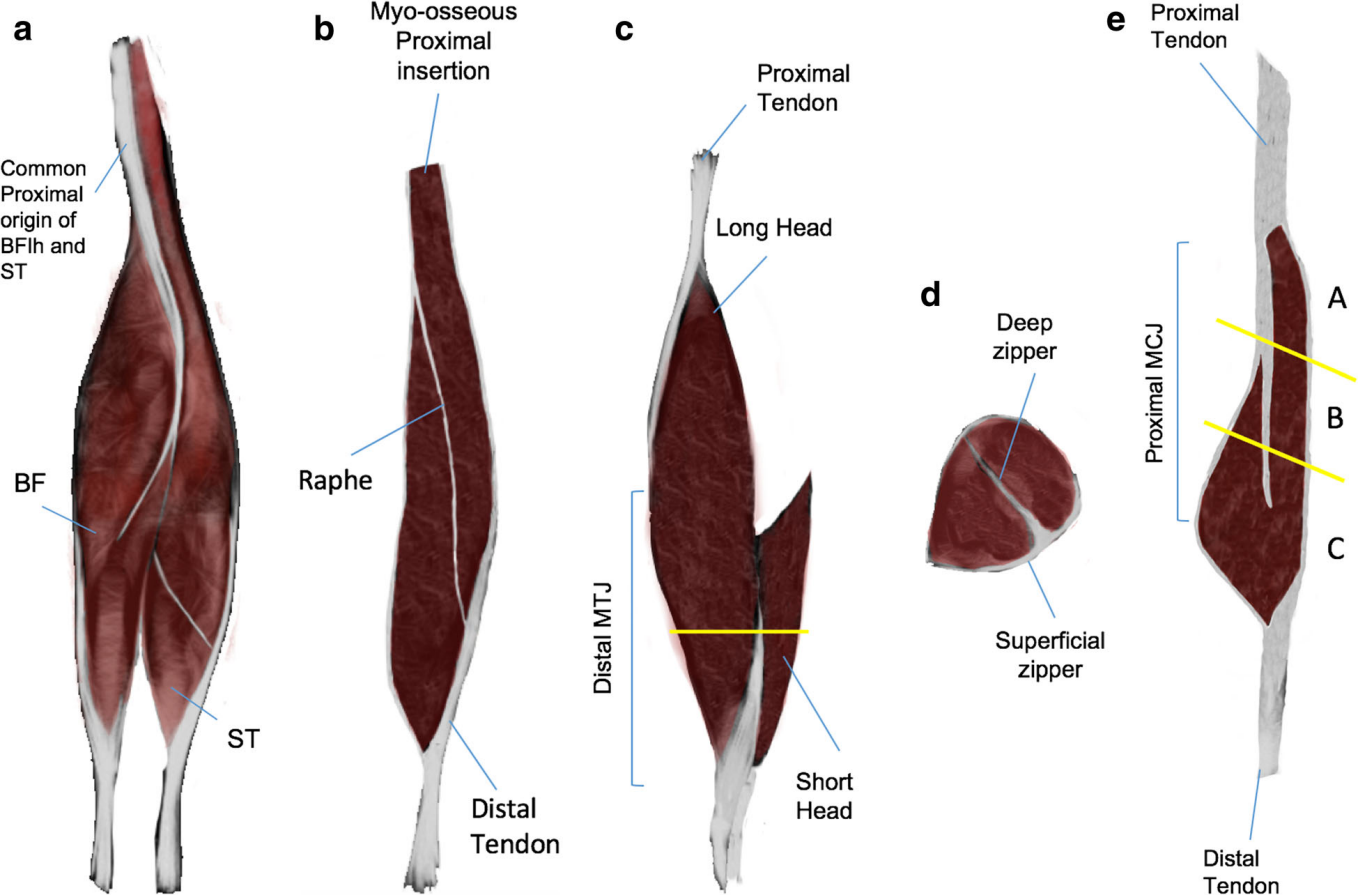
Hamstring complex muscles showing the distribution of connective tissue in each muscle belly. **a** Frontal view of the hamstrings (BFlh, biceps femoris long head; ST, semitendinosus; BF: biceps femoris). **b** Coronal section of the semitendinosus. **c** Open lateral view of the biceps femoris (MTJ, myotendinous junction). **d** Axial section of the distal part of the biceps femoris. **e** Coronal section of the semimembranosus (MCJ, myoconnective junction)

It is a very frequently injured and anatomically very complex muscle group with different thicknesses and distribution of thick connective tissue where the presence of unipennate muscle fibers predominates. The common proximal tendon of the biceps femoris long head (BFlh)/semitendinosus (ST) muscles is found on the posteromedial aspect of the ischial tuberosity (tendinous insertion) (Fig. 1a). In addition to the common origin, muscle fibers of the ST are often seen attaching directly into the ischial tuberosity (myo-osseous insertion) (Fig. 1b). Together, they make up the entire muscle mass of the proximal part of the hamstrings [13]. From its origin, the ST creates a conjoined tendon with the biceps femoris long head (BFlh) forming an aponeurosis (each one his own aponeurosis) [14]. Knowing these particular characteristics helps the radiologist to understand that depending on the location of the lesion, both muscular bellies may be involved; or the BFlh only.

The biceps femoris short head (BFsh) originates from the posterolateral aspect of the femur along the linea aspera. It fuses with the BFlh in the distal part of the thigh, forming an aponeurotic structure (Fig. 1c). The conjoined distal tendon of both heads attaches to the head of the fibula [13]. The distal myotendinous junction (DMTJ) of the biceps femoris has a complex multicomponent anatomy [15, 16] that originates two zippers (superficial and deep) whose location is important (Fig. 1d) given that it has been shown to have a different prognosis [15]. These lesions have a particularly high rate of recurrence, even with prolonged rehabilitation times.

The semimembranosus (SM) has a distinct origin that is separate from, and anterolateral to, the BFlh/ST common tendon origin [14]. Muscles fibers can be classified according to their origin into three sections (Fig. 1e): A (the fibers arise from the medial part of proximal tendon), B (the fibers arise from both parts, medial and lateral part of proximal tendon), and C (bipennate origin, the fibers arise from the distal myoaponeurotic junction) [17]. This differentiation is important to consider the prognosis of the lesions, being worse in sections B and C [17].

### Rectus femoris

**Fig. 2:**
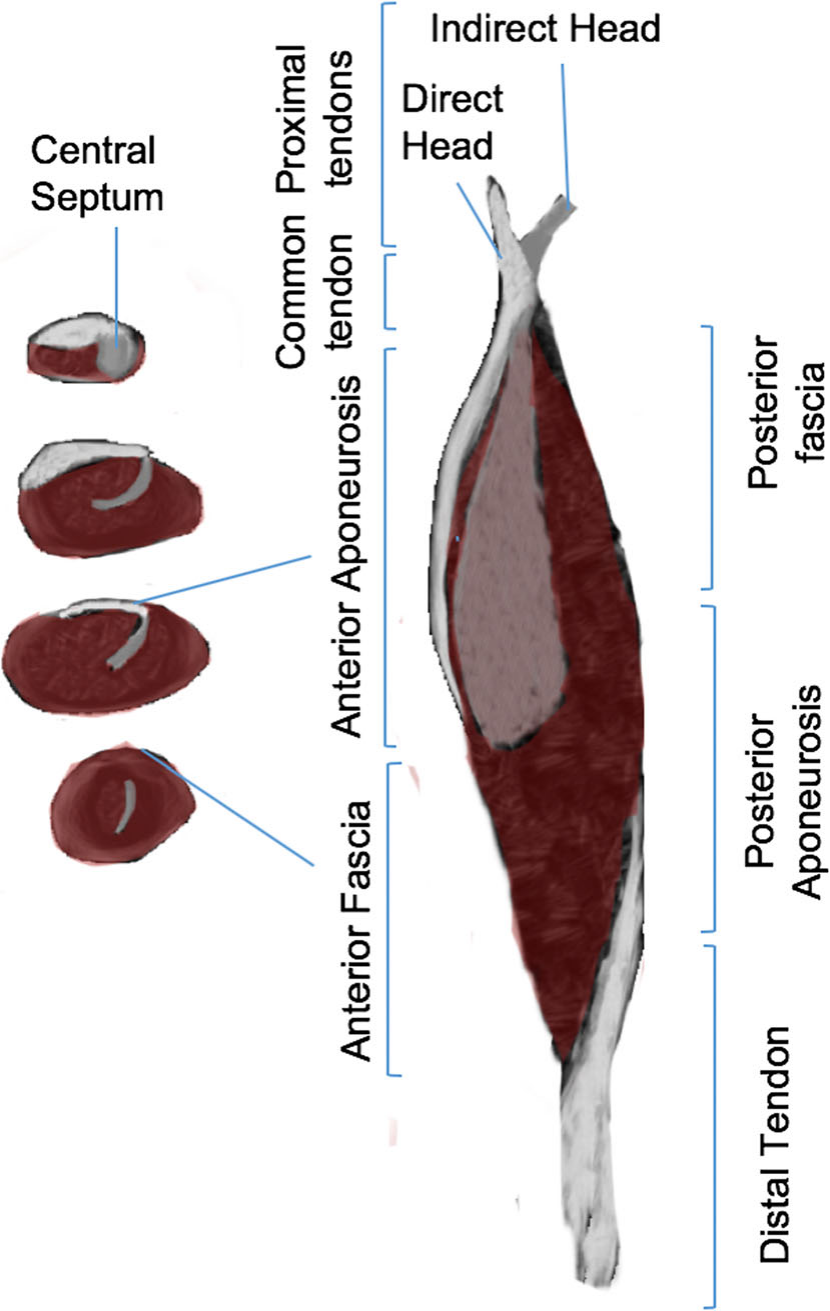
Diagram of the distribution of connective tissue in the rectus femoris. The proximal connective tissue is distributed into the central septum, the anterior aponeurosis, and the anterior fascia; the posterior aponeurosis/fascia is an extension of the distal tendon. Axial sections (left); sagittal section (right)

It is also a frequently injured muscle with a complex distribution of connective tissue (including tendons, central septa, aponeurosis, and fascia) with bipennated disposition fibers surrounding its central portion. The origin of the rectus femoris has two components: a direct tendon (which originates from the anterior inferior iliac spine) and an indirect tendon (which originates from the superior acetabular crest). These two components merge to form a short common tendon [19]. The proximal connective tissue of the rectus femoris is composed of a central septum, aponeurosis, and fascia (Fig. 2). The anterior aponeurosis/fascia is an extension of the proximal direct tendon and the central septum is an (intramuscular) extension of the proximal indirect tendon. From proximal to distal, the connective tissue becomes progressively thinner [19]. The distal connective tissue is composed of a tendon, an aponeurosis, and a fascia. The posterior aponeurosis/fascia is an extension of the distal tendon (Fig. 2). From proximal to distal, the connective tissue becomes progressively thicker [19]. When lesions clinically affect the same muscle level, recognition of the specific structure (e.g., anterior myoaponeurotic, myoconnective of the central septum, posterior myofascial tissues) of the compromised connective tissue by MRI is important to establish the prognosis.

### Adductors

The adductors are composed of the longus, brevis, and magnus. Although the proximal tendon anatomy is complex and there may be common components to the tendon origins, the adductor longus is the most frequently injured of the three. Adductor longus is a fan-shaped muscle, which arises from the anterior aspect of the pubis just inferior to the pubic tubercle and expands to attach to the middle third of the linea aspera of the femur [18, 20]. The proximal tendon is short and conforms an intramuscular aponeurosis. The most frequent tears of the adductor longus are tears of the proximal tendon (including tendon avulsions) or intramuscular midsubstance tears [18, 20]. Distal adductor longus tendon tears are exceedingly rare [18]. On the other hand, the adductor magnus has fibers very close to the hamstring/ischial muscles so that proximal lesions are difficult to identify clinically.

### Calf muscles

**Fig. 3:**
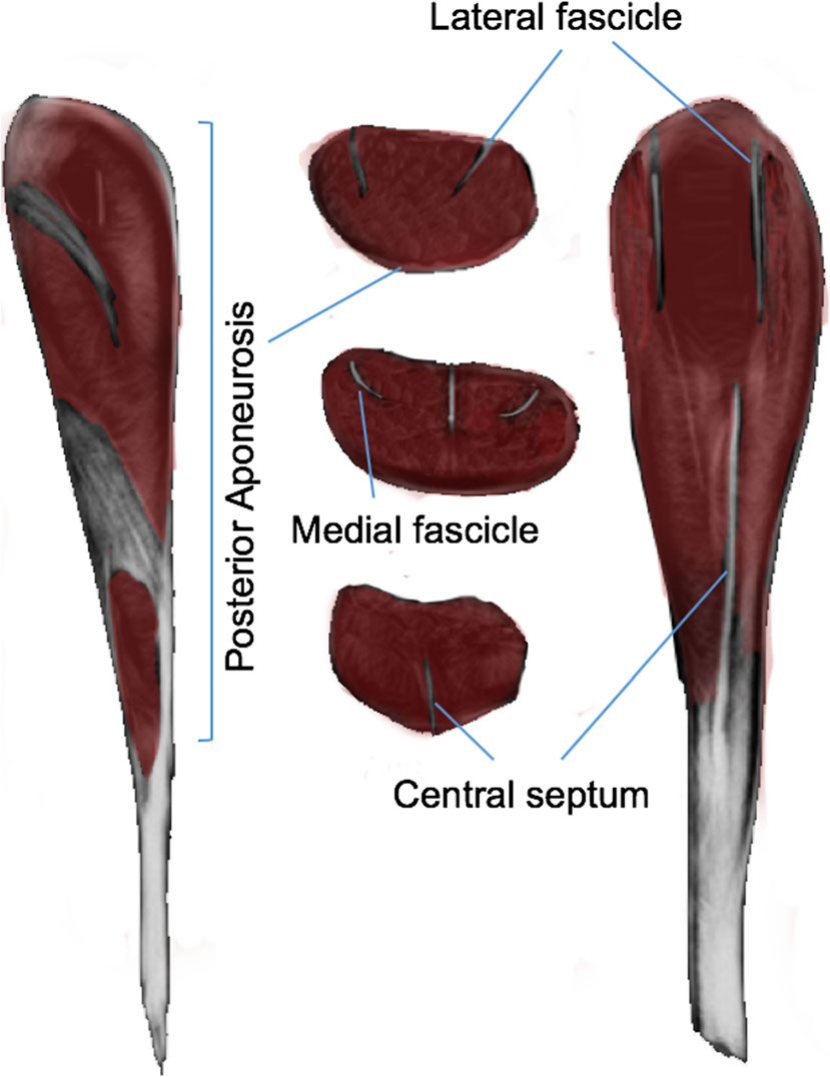
Diagram of the distribution of connective tissue in the soleus identifying medial and lateral fascicles, central septum and aponeurosis (coronal and axial section); only the central septum reaches the posterior aponeurosis (sagittal sections)

The most frequent injuries involving the calf muscles are distal. The distal tendon of soleus inserts together with the tendons of both heads of the gastrocnemius to form the Achilles tendon [6]. The soleus connective tissue architecture has various components, including the medial and lateral fascicles, the central septum, and a posterior aponeurosis (Fig. 3). There is variability in the morphology and arrangement of these structures [6]. The connective tissue that covers the deep surface of the medial gastrocnemius distally blends with the Achilles tendon, resulting in a significant change in caliber which would form a weak point.

## Technical aspects for MRI optimization

MRI has proven to be an essential tool in the assessment of muscle injuries in elite athletes and has rapidly become the imaging modality of choice for the evaluation of the connective tissue [10, 21]. 3 Tesla or optimized 1.5 T MRI allows specialists to define muscle injury with excellent resolution, making acquisitions in the three planes, including oblique planes, and assessing the deep muscles. It is recommended to perform MRI using a multipurpose surface coil to ensure higher resolution images. Protocols should be tailored to ensure thin sections (e.g., less than 4 mm), appropriately small field of view (FOV) (e.g., less than 30 × 30 cm) [11, 22], and multiplanar acquisitions (axial, coronal, and sagittal). The recommended MRI protocol is shown in Table 1. In order to assess extensive muscle areas during an adequate imaging time, pixel sizes of 0.9 × 0.9 mm^2^ can be initially used, achieving pixel sizes of 0.5 × 0.5 mm^2^ with a slide thickness of 1.5 mm for a better characterization of the lesion when necessary. A marker should always be placed to indicate the site of symptoms thus ensuring adequate coverage and, in the case of multiple imaging findings, aiding in determining the clinically relevant lesion. The patient is positioned in supine decubitus and, in general, examination of the injured limb around the area marked with the skin vitamin marker (according to clinical findings) is exclusively performed. This depends in part on the height of the lesion, e.g., for adductors, small and large FOVs are used but focused on the symphysis region.

**Table 1:** 3 Tesla MRI protocol

	Repetition time (TR), ms	Echo time (TE), ms	Section thickness, mm	Interslice distance (GAP), mm	Field-of-view (FOV), cm	Matrix, pixels	Resolution, pixel size mm
Coronal T2-weighted FS	5000	44	2.5	0.6	26 × 29	288 × 320	0.9 × 0.9
Axial T2-weighted FS	5200	44	3.5	0	25 × 25	256 × 256	0.97 × 0.97
Sagittal T2-weighted FS	3700	60	2.8	0	27 × 24	192 × 272	1.4 × 0.88
Coronal T1-weighted	980	11	2.5	0.6	26 × 29	288 × 320	0.9 × 0.9
Axial T1-weighted	900	11	3.5	0	25 × 25	352 × 352	0.71 × 0.71

*FS* fat suppression

The proposed specific MRI protocol is shown in Table 1. It is helpful to perform fluid-sensitive sequences (intermediate-weighted) with an intermediate echo time (TE) (e.g., less than 65 ms) to obtain adequate contrast and spatial resolution of the connective tissues [22]. These sequences enable the detection of edematous changes around the myotendinous, myoaponeurotic, and myofascial junctions, ensuring an accurate connective tissue assessment, as well as allowing the delineation of intramuscular or intermuscular fluid collections or hematomas [10, 11]. T1-weighted sequences are useful in the assessment of subacute hemorrhage or hematoma, in the detection of atrophy and fatty infiltration, and the detection of scar tissue in chronic injuries that may present as chemical shift artifacts in the T2-weighted or fluid-sensitive sequences [10, 11].

## Timing of MRI examination

The assessment of the MRI images should be performed in the context of the clinical diagnosis of the sports physician, the mechanism of injury, the findings of physical examination, and, if performed, the results of other diagnostic imaging modalities such as musculoskeletal ultrasound (US). Likewise, the medical history plays a key role in the interpretation of the lesions. A familiarity with the history of prior injuries (and associated scars) allows more accurate detection and interpretation of new lesions.

The optimal timing of MRI following lower limb muscle injury has not been defined and has been mainly based on expert opinions [12]. In a prospective study of male athletes with acute hamstring injury, there have been no significant day-to-day changes in the extent of edema throughout the first week, with fiber disruption being detectable from the first day after injury [23]. However, the formation of the connective tissue scar starts developing within the first day [24], although changes in the connective tissue scar process can be seen by MRI around the 8th post-injury day [12]. As such, to avoid the accumulation of blood from obscuring the connective tissue, MRI should probably be performed as soon as possible after the injury, within hours if possible.

## MRI findings

### General lesion patterns

**Fig. 4:**
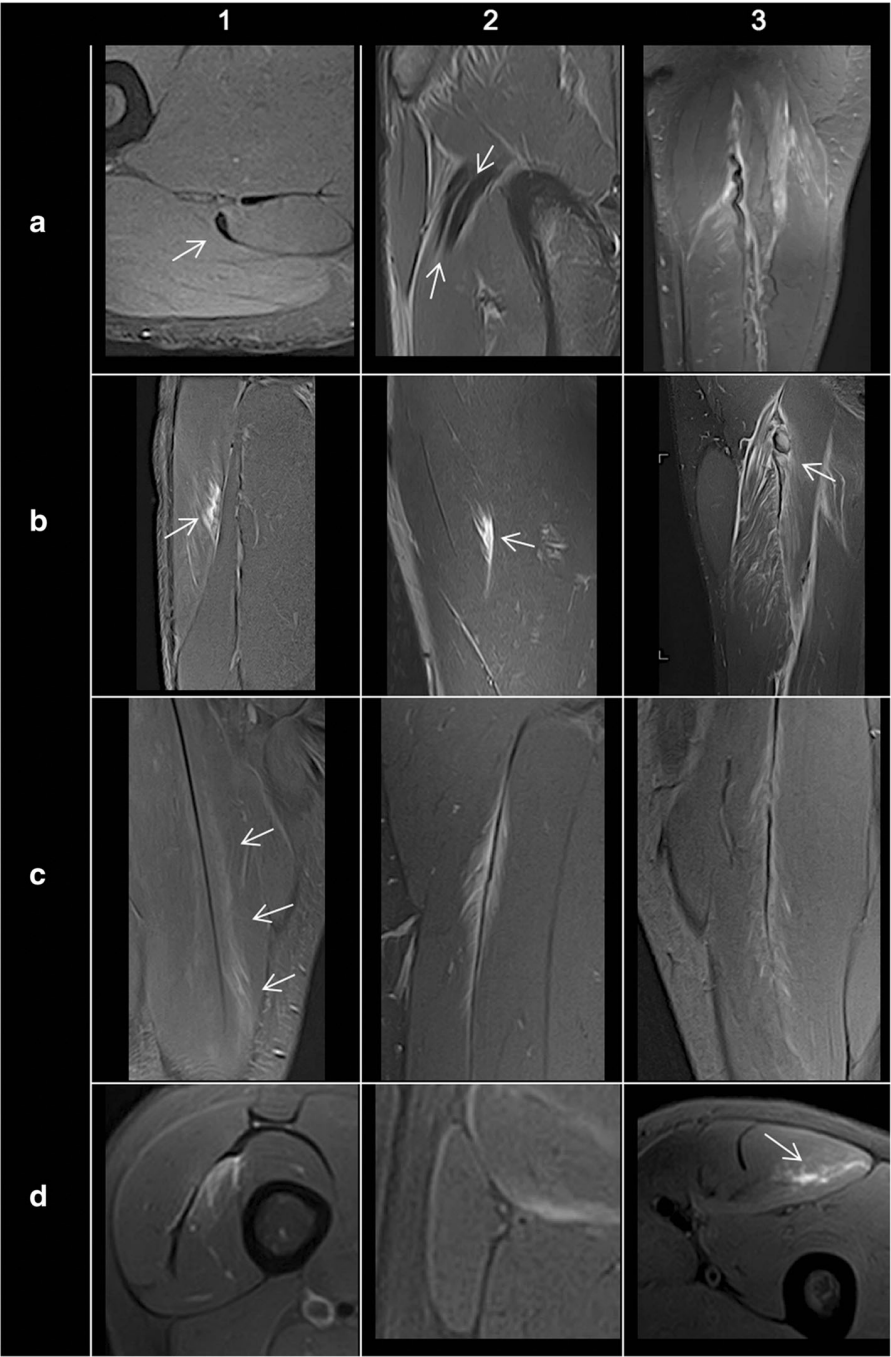
MRI findings and patterns in acute muscle injuries of the lower limb. Tendon injuries showing elongation without a measurable tear (**a**1), longitudinal tear (**a**2), and total transverse tear with marked tendon retraction (**a**3). Peripheral myoaponeurotic/myofascial/myotendinous injuries showing intact aponeurosis (**b**1), fascial tear (**b**2), and tendinous complete section with hematoma and retraction (**b**3). Central myotendinous (septal) injuries showing intact septum (**c**1), fiber gap of the septum (**c**2), and total septal tear with loss of tension (**c**3). Injuries of isolated muscle fibers without disruption (**d**1) with architectural distortion or blurring (**d**2), or with measurable tear (**d**3)

Tendinous injuries (pure connective tissue lesions) may present, in order of severity, as an elongation/stretch injury (slight alteration of intratendinous MRI signal on T1 and/or T2 with subtle halo of peritendinous hyperintensity on T2) (Fig. 4(a1)), a partial tear (Fig. 4(a2)), or a complete tear (hyperintense gap on T2 with clear loss of tendon continuity) (Fig. 4(a3)) [10–12].

The myoconnective junction (MCJ) involves the myotendinous junction and the myofascial/myoaponeurotic junction [9] and is typically involved in indirect muscle injuries. This junction, which is the region of anchorage of the muscle fibers to the connective tissue, is hypointense on all MR sequences, passing through different degrees of thickness (tendon-aponeurosis) until it is no longer evident by imaging (fascia). The MCJ can be located centrally (myotendinous) or peripherally (myofascial/myoaponeurotic) with respect to the muscle belly [9]. In the presence of a disruption of the MCJ, it is very common to identify interstitial edema in a “feathery” pattern (edema that dissects the interstitial space between muscle fibers). This appears as low to intermediate signal intensity on T1-weighted images, and high signal intensity on fluid sensitive sequences [10, 11]. Interstitial edema can appear without muscle fiber disruption (Fig. 4(b1, c1)), with muscle fibers gap (Fig. 4(b2, c2)) or with loss of tension of the connective tissue (Fig. 4(b3, c3)).

On the other hand, muscle fiber injuries can present as architectural distortion (poor definition of fibers on fluid sensitive sequences) (Fig. 4(d2)) or discrete tears (i.e., gap or defect) either at the anchorage point to the tissue connective with consequent loss of the pennation angle or within the muscle belly itself (intramuscular tear, Fig. 4(d3)). In cases of larger tears, intramuscular hematomas may develop.

Intermuscular edema is a very frequent finding that does not necessarily have prognostic value. In direct injuries, the lesions typically affect the muscle bellies adjacent to a bone (e.g., the vastus intermedius along the anterior aspect of the femoral diaphysis; Fig. 4(d1)), and the imaging findings typically affect the muscle fibers (interstitial edema, architectural distortion and even defects), and may or may not be associated with minor connective tissue injury (aponeurosis or fascia). The presence of edema in the adjacent subcutaneous fat is a common finding [11].

Another common type of injury is related to overload, fatigue, or delayed onset muscle soreness (DOMS) where the muscle edema pattern is usually more diffuse and poorly defined (thus we name it cotton-like pattern) [10, 11]. This type of injury rarely results in architectural distortion (Fig. 4(d2)). Also, mild edema-like signal in muscles can be seen in asymptomatic athletes following a training session (post-exercise changes).

### Representative patterns in frequent muscle injuries

**Fig. 5:**
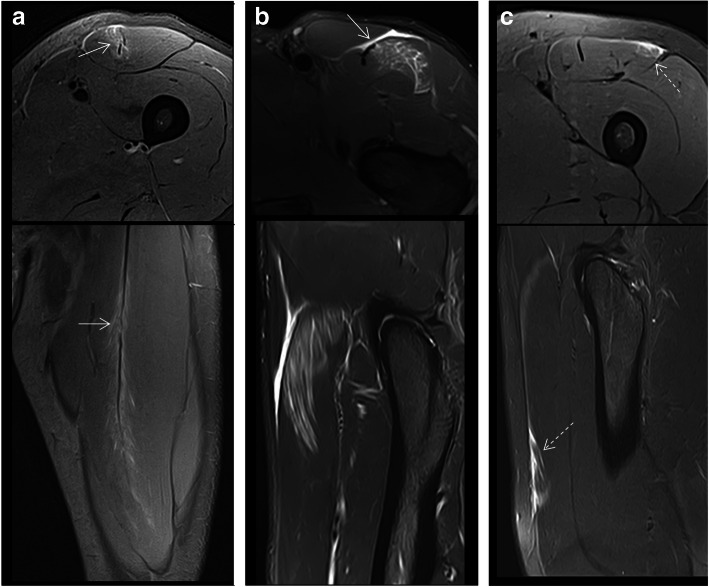
Proximal lesions of the rectus femoris. Fluid-sensitive MRI sequences showed (**a**) gap of the central septum with edema and loss of the pennation angle of the muscle fibers of myoconnective anchor center (axial and coronal images); gap of the aponeurosis (arrows) with interfascicular and intermuscular hemorrhage (**b**) (axial and sagittal images); discontinuity of the connective tissue of lesser thickness (dashed arrows) with peripheral fluid and fiber gap in the anchorage of peripheral myoconnective fibers (**c**) (axial and sagittal images)

**Fig. 6:**
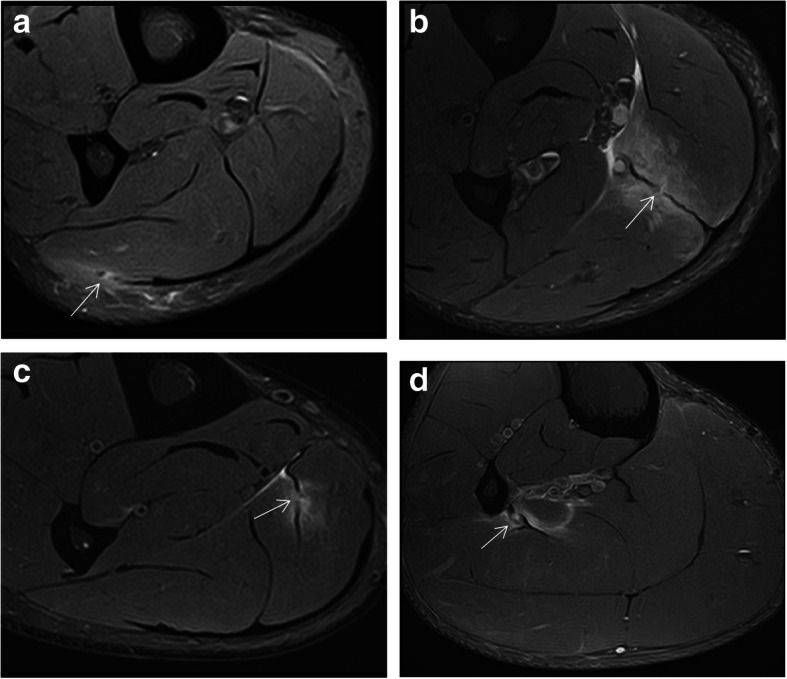
In the soleus, axial MR images in fluid-sensitive sequences of four different patients show edema surrounding the connective tissue injured and its discontinuity (arrows) involving different structures: (**a**) the posterior aponeurosis, (**b**) central septum, (**c**) medial fascicle, and (**d**) lateral fascicle

**Fig. 7:**
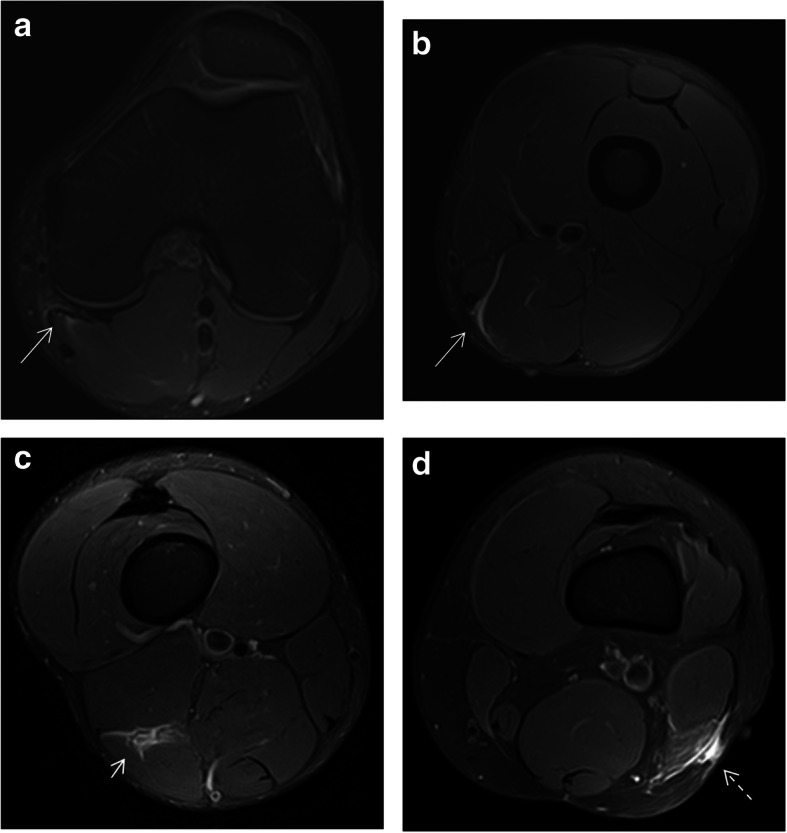
Axial MR images in fluid-sensitive sequences in distal myoconnective injuries of the semimembranosus muscle showing muscular edema (arrows) at different levels (**a** and **b**). Edema in the deep (short arrow, **c**) and superficial (dashed arrow, **d**) zippers of the femoral biceps

Lesions of the rectus femoris include gap of the central septum (Fig. 5a), gap of the anterior aponeurosis (Fig. 5b), and discontinuity of the anterior fascia (Fig. 5c). In the clinical cases shown in Fig. 5, return to sport was longer in central septum and anterior aponeurosis lesions (6–7 weeks) compared to the anterior fascial injury (2–3 weeks). In the soleus, main myoconnective tissue injuries include rupture of the posterior aponeurosis (Fig. 6a), central septum (Fig. 6b), medial fascicle (Fig. 6c), and lateral fascicle (Fig. 6d). Lesions affecting the central septum and the medial fascicle are usually associated with a longer return to sport (5–6 weeks) than the remaining lesions (3–4 weeks). Examples of lesions of the semimembranosus and biceps femoris zippers are shown in Fig. 7. Return to sport may be expected to be relatively short (3–4 weeks) in less extensive myotendinous injuries of the semimembranosus (Fig. 7a) and lesions of the deep zipper of the femoral biceps (Fig. 7c).

**Fig. 8:**
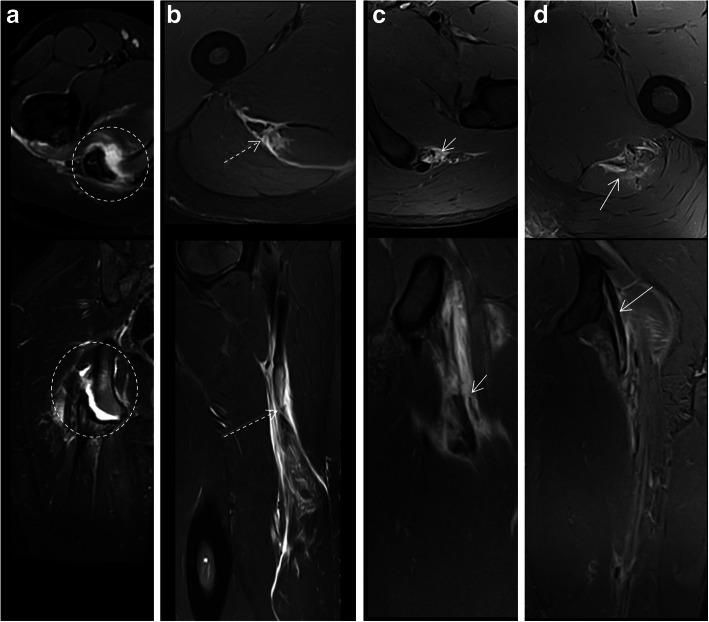
Axial and coronal/sagittal MR images in fluid-sensitive sequences of the proximal hamstring complex tendon. **a** Complete apophyseal avulsion fracture of the right ischial tuberosity with severe increase of the signal intensity at the avulsion site (dashed circles). **b** Complete transverse tear (dashed arrows) of the common tendon of the biceps femoris and the semitendinosus with thickening and distal retraction. **c** Complete avulsion of the semimembranosus proximal tendon (short arrows) with retraction. **d** Mixed partial rupture of the semimembranosus proximal tendon

**Fig. 9:**
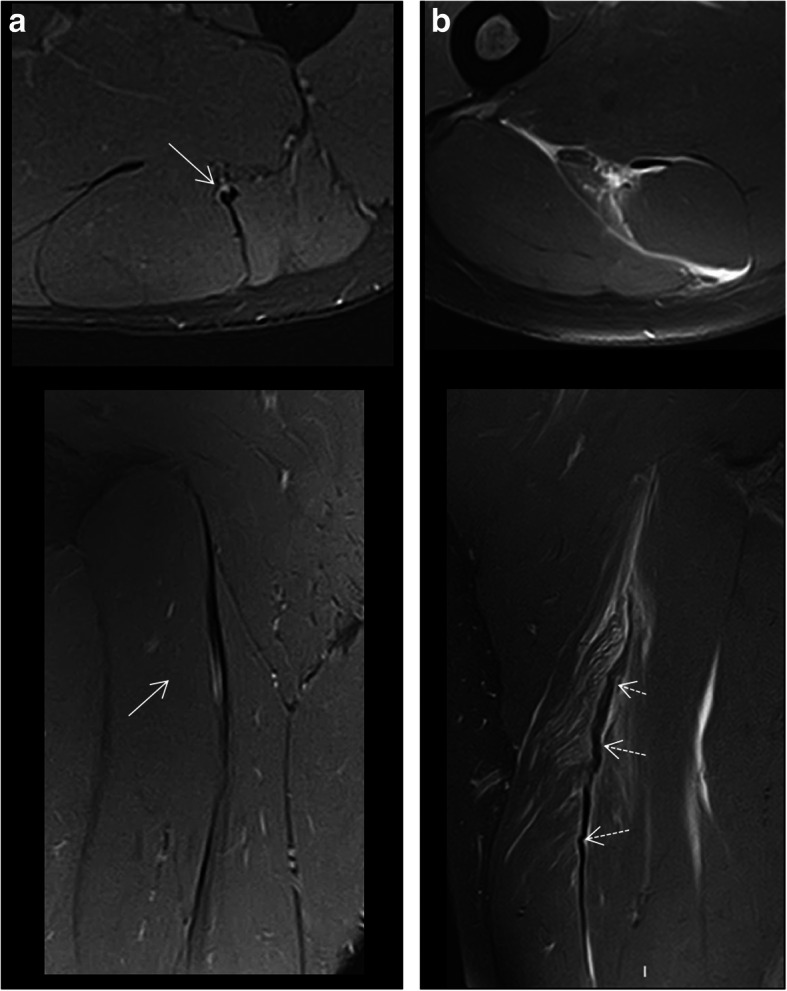
Coronal and axial MR images in fluid-sensitive sequences of the long head of the biceps femoris tendon. **a** Partial mixed disruption (arrows), predominantly longitudinal without loss of tendon tension. **b** Complete disruption with loss of tendon tension (wavy morphology, dashed short arrows), extensive interstitial edema, and intermuscular fluid

In the hamstring complex, proximal tendon injuries include insertional (Fig. 8) or non-insertional (Fig. 9) ruptures. Tears may be transverse (Fig. 8b, c), longitudinal (Fig. 9a), or mixed (depending on the orientation of the tear with respect to the long axis of the tendon) (Fig. 8d). When a significant cross-sectional area of a tendon is injured, there can be loss of tendon tension which manifests on imaging as a wavy tendon (Fig. 9b). In cases where there is connective tissue scarring due to a previous injury, the sign of loss of tension (i.e., wavy tendon) may not be observed since the tendon may be stiffer or may be anchored/tethered by scar tissue. In addition, the patient’s age is an important factor in assessing tendon-related lesions. In children, adolescents, and skeletally immature young adults, the physeal cartilage is not calcified and is thus a weak point. This makes apophyseal and physeal traction injuries more common that true tendon injuries. In mild cases, MRI can show bone marrow edema of the apophysis on fluid-sensitive sequences without signal changes in the physis or displacement of the apophysis (so-called isolated apophysitis or apophyseal stress injuries) [25]. In more advanced or severe cases, MR may show fluid signal in the physeal cartilage, and finally, widening of the physeal cartilage with separation (Fig. 8a) and even displacement of the apophysis in the case of apophyseal avulsions [25].

**Fig. 10:**
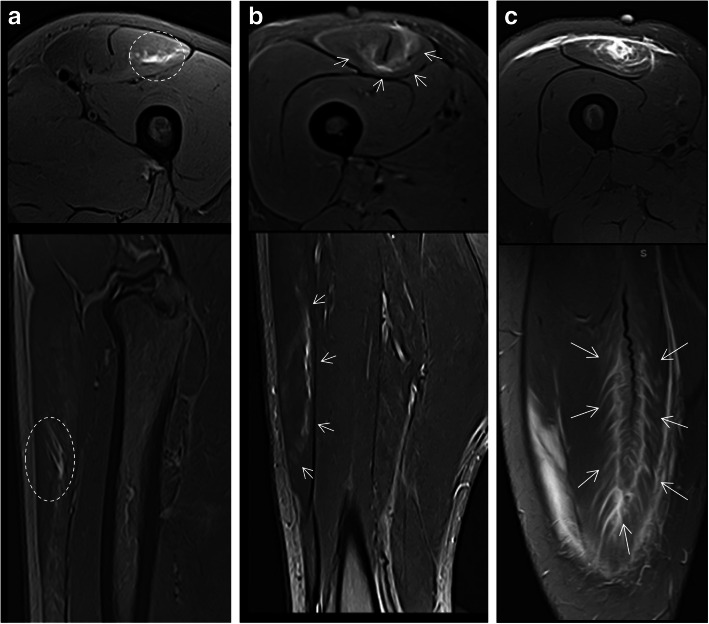
Intramuscular isolated injuries of the rectus femoris. Axial and coronal/sagittal MR images in fluid-sensitive sequences. **a** Marginal or paraseptal muscular edema with gap of the muscle fibers (circles). **b** Edema, fiber distortion, and areas of tear surrounding the central septum (short arrows) without retraction. **c** Circumferential pattern of fibers rupture encompasses the entire central septum with loss of its tension (large arrows)

Figure 10 shows examples of intramuscular isolated injuries of the rectus femoris, in which the central septum is usually preserved (Fig. 10a, b). However, indirect retraction of the central septum may be present according to the location of the intramuscular tear (Fig. 10c).

## MRI classification of muscle injuries

To date, classification systems of muscle injuries have been based on US and MRI findings. In the past 7 years, three muscle injury classifications have been reported, including the Munich consensus statement [3], the British Athletics Muscle Injury classification [4], and the FC Barcelona-Aspetar-Duke classification [5], the main characteristics of which are shown in Table 2. There is no agreement between classification systems in the differentiation of the type of tissue involvement (muscle fibers, tendon, aponeurosis), something that may be of great importance. Two of the classification systems mostly used by physicians do not employ such a strict quantification of the observed findings [3, 5]. However, in order to categorize the lesions according to their severity and prognosis, the British Athletics Muscle Injury suggests that these findings should be measured. Also, some classifications [4, 5] consider the measurement of interstitial muscular edema which can be quantified according to cross-sectional area or cranio-caudal length, whether it is associated with blurring or not [4]. Additionally, the blurring or architectural distortion area can be also measured [4]. However, only the FC Barcelona-Aspetar-Duke classification [5] includes the mechanism of action of the traumatic event and its location in relation to the entire muscular belly, whereas the Munich consensus statement [3] is the only system that includes fatigue-induced muscle disorder and DOMS. In all three classification systems, the involvement of the thick connective tissue (tendon) especially if there is retraction is considered to be the worst prognostic sign; this is typically manifested clinically as loss of function. Apophyseal avulsion injuries are not taken into account in any of the classification systems.

**Table 2:** Summary of muscle injury classification systems

Munich Consensus Statement [3]		British Athletics Muscle Injury Classification [4]		FC Barcelona-Aspetar-Duke classification [5]	
Grade 1 (fatigue-induced muscle disorder)	Negative MRI imaging/US findings	O a/b	MRI imaging normal/MRI imaging normal or patchy edema	Mechanism of injury (M)	I (indirect) T (direct)
Grade 1B (delayed-onset muscle soreness, DOMS)	Negative MRI imaging/US findings or edema only	1a (small myofascial tear)	Edema at the fascial border, <10% extension into muscle belly; <5% CC length	Location of injury (L)	P (proximal) M (middle) D (distal)
Grade 2A (spine-related neuromuscular muscular disorder)	Negative MRI imaging/US findings or edema only	1b (small muscle-tendon junction tear)	< 10% of CSA of muscle the MTJ; < 5 cm CC length; may note fiber disruption of < 1 cm	Grading of severity (G)	0 (negative MRI)
Grade 2B (muscle-related neuromuscular muscle disorder)	Negative MR imaging/US findings or edema only	2a (moderate myofascial tear) 2b (moderate muscle-tendon junction tear)	Edema evident at fascial border with extension into the muscle or edema evident at the MTJ; Edema of CSA between 10–50% at maximal site; CC length 5–15 cm; architectural fiber disruption < 5 cm		1 (edema without intramuscular hemorrhage or architectural distortion)
Grade 3A (minor partial muscle tear)	Fiber disruption on 1.5 or 3-T MR images; intramuscular hematoma	2c (moderate intratendinous tear)	Edema extends into the tendon with longitudinal length of tendon involvement (< 5 cm); < 50% of maximal tendon CSA. No loss of tension or discontinuity within the tendon		2 (edema with minor muscle fiber architectural distortion ± minor intermuscular hemorrhage, but no gap between fibers)
Grade 3B (moderate partial muscle tear)	Significant fiber disruption, probably including some retraction, with fascial injury and intermuscular hematoma	3a (extensive myofascial tear) 3b (extensive muscle-tendon junction tear)	Edema at the fascial border with extension into the muscle or at the muscle-tendon-junction; > 50% CSA at the muscle-tendon-junction; > 15 cm CC distance; architectural fiber disruption > 5 cm		3 (any quantifiable gap between fibers with partial retraction ± intermuscular hemorrhage)
Grade 4 (subtotal/total muscle tear or tendinous avulsion)	Subtotal/complete discontinuity of muscle/tendon; possible wavy tendon morphology and retraction, with fascial injury and intermuscular hematoma	3c (extensive intratendinous tear)	Edema extends into the tendon with longitudinal length of tendon involvement > 5 cm; > 50% of maximal tendon CSA; probably loss of tendon tension, no discontinuity	Tendinous lesion (r)	r (presence or not depends of tendinous involvement)
		4 (full thickness tear of muscle)	Complete discontinuity of the muscle with retraction	Number of muscle re-injuries (R)	0: 1st episode 1: 1st re-injury 2: 2nd re-injury

*CC* circumferential, *MTJ* musculotendinous junction, *CSA* cross-sectional area

The limitation of all of these classification systems is that the complexity and variability of the overall architecture of different muscles makes generalization of prognosis difficult, if not impossible. Furthermore, consideration of the relationship between the injured muscle, its specific function and athlete’s sport (and position) is crucial in determining the severity and implications of the injury. Despite the limitations of the classification systems, a code-based categorization of muscle injuries provides a common language and ensures clear and accurate communication between all health care professionals involved (sports medicine physicians, radiologists, orthopedists, physiotherapists, etc.).

## MRI reporting

There should be a standardized method of reporting muscle injuries. This will ensure consistency not only in communicating characteristics of the present injury but also in the follow-up of injuries or re-injuries. Although there is no single perfect standardized method of reporting, the following approach can be recommended. First of all, one should describe the location of the pathological findings in relation to the muscle (proximal, middle, or distal) and in relation to the MCJ (proximal or distal). Then, it is most effective to describe the signs in decreasing order of probable prognostic significance. The status of the tendon is described first (measurable gap, tendon retraction, or loss of tendon tension), then the MCJ (loss of the pennation angle, gap of muscle fibers, blurring of them, and interstitial edema) and thirdly, the thinner MCJs centered on the aponeurosis and fascia (measurable gaps). Next, isolated muscular edema, intra- or intermuscular hematomas, and intermuscular fluid are reported; these are not routinely measured, although doing so may be desirable in some situations. Finally, the FCB-Aspetar-Duke classification is used to ensure clear and consistent communication of the imaging findings to all the health care professionals involved with the hopes of having a more reliable and reproducible sense of the prognosis of each injury.

## Healing and post-injury MRI assessment

It is important to know the MRI features of the muscle injury but it is perhaps as important to know the appearance of the healing process in order to be able to identify potential complications. The reparative phase of an injury starts on days 2 and 3, consisting of phagocytosis of necrotic tissue, concomitant production of a connective tissue scar accompanied by capillary ingrowth at the location of the injury, and an activation of satellite cells that will differentiate into myoblasts which are ultimately responsible for the renewal of the skeletal muscle [24]. An inflammatory response associated with edema at the location of the injury and its surroundings is observed shortly after injury. For this reason, mild persistent peritendinous edema may be considered reparative edema.

**Fig. 11:**
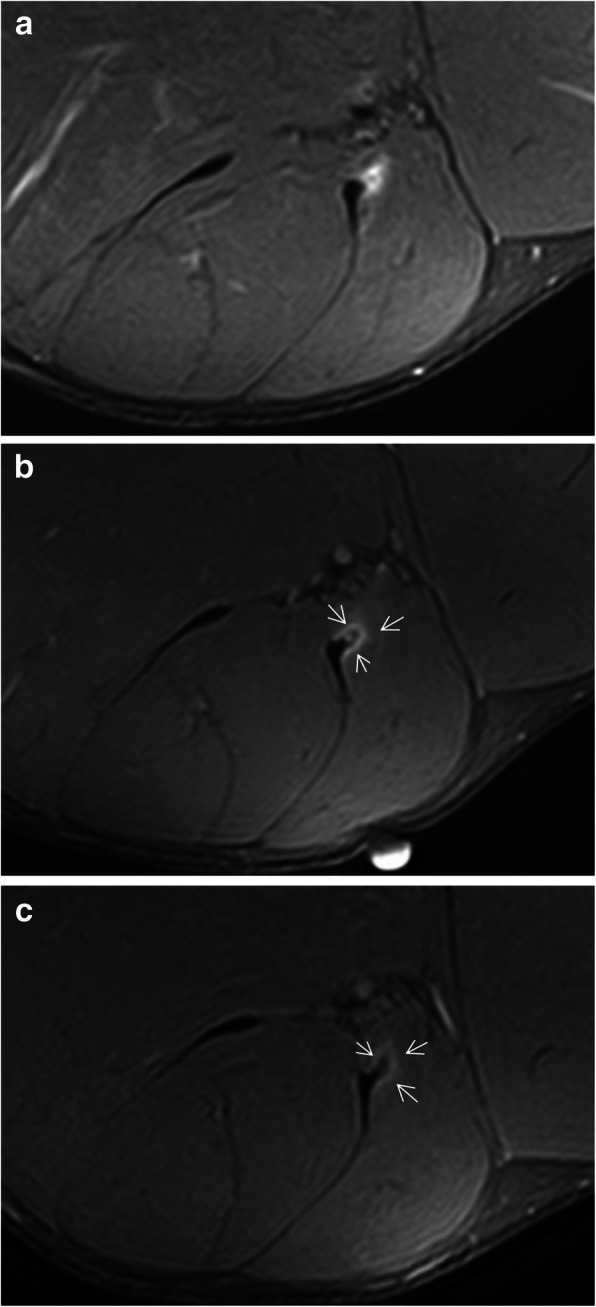
Evolution of healing by MRI over a course of 15 days: hamstring myotendinous junction. Axial fluid-sensitive images at 24 h (**a**), 8 days (**b**), and 15 days (**c**). At 8 days, the soft scar is present with centripetal growth although with reparative pericicatricial edema that remains at 15 days (**b** and **c**, arrows)

**Fig. 12:**
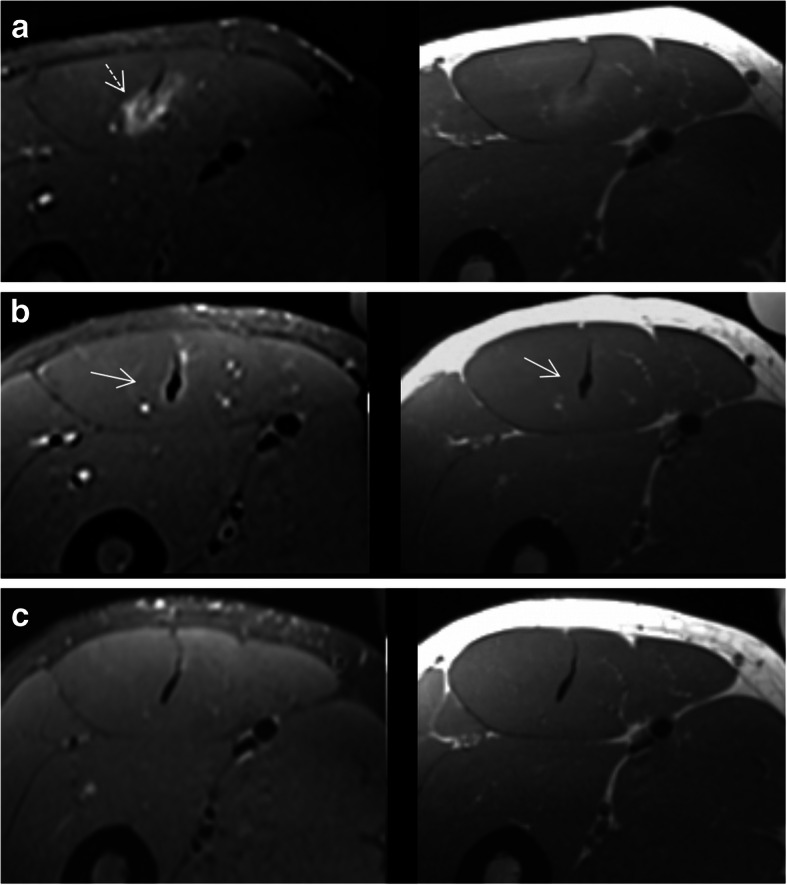
Evolution of healing by MRI over a course of 12 weeks: rectus femoris central myoconnective junction. Axial T1- and fluid-sensitive images at 1–2 weeks (**a**), 9 weeks (**b**), and 12 weeks (**c**) show progressive reduction of edema. **a** MR images show a soft fragmented callus (dashed arrow). **b** MR images show the callus organized and complete. It is hypertrophic, hypointense in fluid-sensitive image, and slightly heterogeneous in T1 (arrows). **c** MR images show a complete recovery of the tendon and peripheral musculature signal

**Fig. 13:**
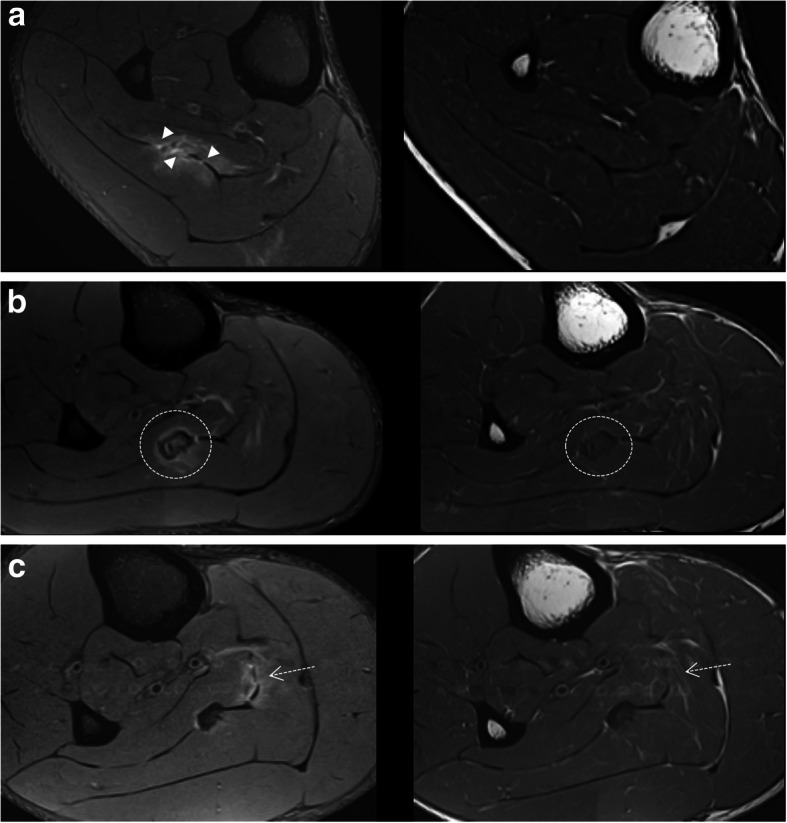
Evolution of healing by MRI over a course of 8 weeks: soleus with common aponeurosis, a normal anatomical variant, myoconnective junction. Axial T1- and fluid-sensitive images at 24 h (**a**), 4 weeks (**b**), and 8 weeks (**c**). **a** MR images show an extensive initial lesion with several foci of rupture of the lateral portion of the common aponeurosis (arrowheads). **b** MR images show an hypertrophic soft scar (dashed circle). **c** MR images show a medial re-injury in the common aponeurosis (dashed arrow)

The healing process over a period of 12 weeks in three different muscles is shown in Figs. 11, 12, and 13. In the first 2 weeks, a decrease in fluid-like signal intensity at the location of the injury (Fig. 11) can be observed on MRI [26]. A “soft” scar tissue is observed as early as 8 days post-injury [24], which shows a T2 hyperintense center surrounded by a very thin hypointense peripheral line (Figs. 11b and 12a). T1-weighted images do not seem to provide relevant information in this phase. At 15 days, if there is good reparative progress, the extension and high T2 signal of the soft tissue edema is greatly reduced (Fig. 11c). At the same time, the gap of connective tissue should appear more heterogeneous and with lower T2 signal. In this phase, the scar, being relatively soft, can appear fragmented (Fig. 12a). If there is feathery peritendinous interstitial edema, overload can be suggested. Alternatively, the identification of a DOMS type edema pattern appears to be a sign of adaptation and good prognosis.

At 6 weeks, the hypointense peripheral line grows centripetally and the callus signal is more hypointense and homogeneous (and possibly thicker) on T2-weighted images, and intermediate to low signal intensity on T1-weighted images (Fig. 13) [27]. At 8–10 weeks, T2-weighted images show further hypointensity and T1-weighted images show very low signal intensity (Fig. 13c). At 12–14 weeks, the tendon shows a homogeneous hypointensity on both T1- and T2-weighted images, and its morphology becomes almost normal with some thickening or irregularity/nodularity due to scarring [27].

**Fig. 14:**
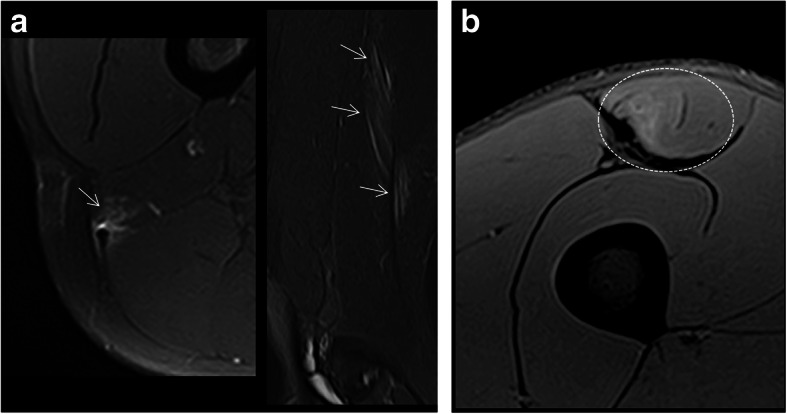
Muscle signal changes in muscle fibers, adaptive or by overload, in the recovery from distal myoconnective injury of the femoral biceps and the rectus femoris. **a** Axial and sagittal fluid-sensitive images show areas of hyperintensity of muscle fibers adjacent to scar in the form of feathery peritendinous interstitial edema (arrows) suggesting overload. **b** Axial fluid-sensitive image shows area of slightly hyperintensity of muscle fibers adjacent to scar in the form of cotton-like edema pattern (DOMS, dashed circle) suggesting adaptation

Finally, it is of utmost importance to comment on the appearance of any interstitial edema on follow-up MRIs to guide the conditioning training and recovery of the player. If at 4–6 weeks post injury there is feathery edema around the soft scar (Fig. 14a), it is a suggestion of overload and the intensity of physical training is thus reduced. In contrast, physical recovery and training should continue with the same load if cotton-like pattern edema (DOMS pattern) is observed since this appears to be an adaptive sign (Fig. 14b). If no edema is observed, the load of the training sessions may be increased.

## MRI findings and return to play

In the setting of elite athletes, MRI is considered the imaging modality of choice for predicting the moment to return to play after acute muscle injury, together with other player-related factors (e.g., position of the player in the team, time of the season, etc.). Previous research has suggested that a number of imaging findings are associated with the time elapsed before returning to play [28–31]. Connective tissue involvement is related to prolonged return to sport, particularly if it is a central or proximal tendon (free tendon) or near the tendon origin, and especially if there is retraction or loss of tension [32–35]. Injuries without hyperintensity on fluid-sensitive sequences are associated with a shorter time to return to sport [36]. The extension of muscular edema, the length of lesions, or the presence or absence of intramuscular hematomas are not as clearly related to time to return to sport [36, 37].

However, the issue of return to play is complex. According to a recent systematic review, there is no strong evidence for any MRI finding for predicting the time away from a sports practice after a player has suffered a hamstring injury [32]. Cruz and Mascarenhas [38] published an excellent review of adult thigh injuries and summarized the prognostic value of MRI findings described in different studies. Interestingly, the evidence level of individual MRI features is generally limited, conflicting or moderate, probably because the beginning of sports practice is multifactorial and also relies on clinical factors determined by the player as well the sports physician and the physiotherapist. In a study of 176 athletes with acute hamstring injury, the presence of overlapping or variation within the classification systems (modified Peetrons, Chan acute muscle strain injury classification, and British Athletics Muscle Injury Classification) did not allow to predict return to sport in this population [39]. Dimmick and Linklater [40] published an in-depth review of acute hamstring muscle strain injuries and also concluded that imaging grading systems have a limited utility in predicting return to play. Two studies that focused on the relationship between MRI features and return to play [36, 41] revealed the persistence of an increased signal intensity on fluid-sensitive sequences at the time of the clearance to return to sport. This suggests that functional recovery precedes signal normalization at imaging. As such, normalization of the signal on MRI images does not appear to be a requirement for clearance to return to play.

## Stepwise systematic approach

A summary of stepwise systematic approach for MRI assessment of muscle injuries in elite sport is as follows:

1. Clinical information: verify date of trauma, mechanism of injury, symptoms, and sports discipline. Verify history of prior injuries in the same region.
2. Evaluation of the MRI study

- Anatomical assessment on T1-weighted sequences (axial and coronal): individual muscular anatomy, anatomical variants, residual changes from previous lesions (scarring, atrophy), and vascular structures.
- Lesion assessment on T1 and T2/fluid-sensitive sequences (acute lesions on T2-weighted sequences and previous lesions on both T1- and T2-weighted sequences): location of the lesion (proximal, middle, distal), anatomical structures involved (aponeurosis, fascia, tendon, fibers), and pattern of edema and/or scar.

3. Categorization of the MR lesion based on clinical and imaging criteria.

## Conclusions

Several imaging classification or grading systems for muscle injuries, especially strains, are available for application in clinical practice and clinical research. The widely used grading system for muscle injuries (grades 1–3) provides limited prognostic information to sports medicine physicians, since it does not properly cover the full spectrum of muscle injuries. The consideration of the specific anatomical characteristics of each muscle group is essential for a proper MRI diagnosis and clinical approach of individual athletes. Standardized radiological reporting and classification/staging of lesions in association with other clinical factors allow physicians to more accurately assess the severity of an injury, as well as to establish a treatment plan, recovery program and to estimate the expected time to return to sport. Although MRI is a good tool for assessing the healing process, it is important to keep in mind that functional recovery precedes normalization of MRI signal abnormalities. Studies focused on the MRI characteristics of the healing process are a promising line of future research.

## Data Availability

Data sharing is not applicable to this article as no datasets were generated or analyzed.

## Abbreviations

BFlh: Biceps femoris long head
BFsh: Biceps femoris short head
DMTJ: Distal myotendinous junction
DOMS: Delayed onset muscle soreness
FOV: Field of view
FS: Fat suppression
MCJ: Myoconnective junction
MRI: Magnetic resonance imaging
SM: Semimembranosus
ST: Semitendinosus
TE: Echo time
TR: Repetition time
US: Ultrasound
